# Editorial: Advances in Analytical Techniques and Methodology for Chemical Speciation Study

**DOI:** 10.3389/fchem.2021.692144

**Published:** 2021-05-10

**Authors:** Ottavia Giuffrè, Anna Napoli, Carlos Rey-Castro

**Affiliations:** ^1^Dipartimento di Scienze Chimiche, Biologiche, Farmaceutiche ed Ambientali, Università di Messina, Messina, Italy; ^2^Dipartimento di Chimica e Tecnologie Chimiche, Università della Calabria, Arcavacata di Rende, Italy; ^3^Departament de Química, Universitat de Lleida and AGROTECNIO-CERCA, Lleida, Spain

**Keywords:** speciation and sequestering ability, remediation techniques, chelation therapy, modeling and computational approach, sensors

On the basis of the International Union for Pure and Applied Chemistry (IUPAC) definition “speciation analysis” involves those “analytical activities of identifying and/or measuring the quantities of one or more individual chemical species in a sample”. “Speciation of an element” is also defined as “distribution of an element amongst defined chemical species in a system” (Templeton et al., 2000; Templeton and Fujishiro, 2017). It is now established that speciation studies in aqueous media are crucial for assessing species toxicity and bioavailability, biogeochemical cycling, and many biological phenomena. Novel studies in this field include a multidisciplinary approach *via* the use of different experimental analytical techniques as well as their combination with simulation methods.

Several examples of speciation of metal cations in aquatic environment are reported in this Research Topic. The speciation of trace metals aquatic systems includes the determination of free ions, metal complexes, colloidal species, etc., as well as the total dissolved concentration. The integrated assessment of free ions and labile metal complexes can be obtained by Diffusive Gradients in Thin-films (DGT), a dynamic speciation technique. The determination of the organic pools of trace metals in freshwaters and the characterization of organic and inorganic complexes in sea waters were obtained by this procedure (Galceran et al.). For organic Cu speciation, an improved anodic stripping voltammetry (ASV) method, employed to eliminate the surface-active substances (SAS) interference on the voltammetric signal, was used in samples containing high organic matter concentration. Fulvic acid was used as a model of natural organic matter and the method was applied for Cu speciation in samples collected in the Arno River estuary (Italy) (Padan et al.). The binding of Cd(II), Pb(II), and Zn(II) by silica nanoparticles was studied using a combination of the electroanalytical techniques Scanned Stripping ChronoPotentiometry (SSCP) and Absence of Gradients and Nernstian Equilibrium Stripping (AGNES). The experimental system was chosen as a representative model for the role of natural and anthropogenic nanoparticles in the fate and behavior of trace metals in aqueous environmental systems (Rotureau et al.).

Speciation studies on the interaction of metal cations with ligands having multiple binding sites, such as tannic acid, a natural polyphenolic compound, and phytate ligand, are very important for the fate of those metals. In the study regarding tannic acid, the approach used combines UV-vis and fluorescence spectroscopy with chemometrics, namely Multivariate Curve Resolution-Alternating Least Squares (MCR-ALS) and Parallel Factor Analysis (PARAFAC) (Berto and Alladio). The investigation of the interaction of phytate ligand with biologically relevant cations, namely Mg^2+^, Zn^2+^, Fe^2+^, Cu^+^, and Cu^2+^ revealed that alkaline earth metals interact with different binding sites than the transition metals. Experiments with Cu^+^, and Cu^2+^ confirmed similar complexing behaviors, depending mainly on the ionic radius (Marolt et al.).

Metal speciation can be also performed by Electrospray Ionization Mass Spectrometry (ESI-MS). In this field, MS-MS approach guarantees exceptional sensitivity, as well as the use of high-resolution sectors, capable of well-resolving isotopic clusters. A further development is the merging of ESI-MS information with data obtained *via* synergistic techniques, as ICP-MS, NMR, X-RAY, CLE-ACSV, Ab-Initio, or DFT quantum mechanical calculations (Indelicato et al.). The necessary degree of specificity in discriminating all the species of a given element in a sample can be attained by ultrahigh-resolution mass spectrometry based on the Fourier transformation, Orbitrap and Ion Cyclotron Resonance (ICR) cell. The case of the speciation analysis of the products of selenium metabolism by FT ICR MS was described (Bierla et al.). Metal speciation can be also performed by MALDI-MS and tandem mass spectrometry (MS-MS). The results obtained for Ca^2+^ interaction with biologically relevant ligands, as cysteine, D-penicillamine, reduced glutathione, and oxidized glutathione obtained by potentiometry and ^1^H-NMR spectroscopy were confirmed by MALDI-MS and MS-MS, elucidating also the mechanism of interaction (Aiello et al.).

Speciation studies also include the search for novel drug delivery systems able to improve the performance of old-generation antibiotics. For example, the capability of two micellar polycationic calix[4]arene derivatives to recognize and host ofloxacin, chloramphenicol, or tetracycline in aqueous solution was investigated by nano-isothermal titration calorimetry, dynamic light scattering, and mono- and bi-dimensional NMR. Results evidenced that the formation of the chloramphenicol–micelle adduct is enthalpy driven, whereas entropy drives the formation of the adducts with both ofloxacin and tetracycline. NMR spectra confirmed ITC data about the positioning of the antibiotics in the calixarene nanoaggregates (Migliore et al.).

Speciation studies are also very useful for the development of techniques for the decontamination of natural waters and soils containing toxic metals. In recent years, many efforts have been made to discover new technologies that are effective, robust, cost-effective and easy to handle for the decontamination of downstream water without endangering human health. Among nanomaterials and nanostructures proposed in the remediation field, graphene-based materials (G), are particularly suitable for the development of reliable water decontamination treatments, in particular for arsenic remediation (Foti et al.). Different methodologies are employed for soil remediation. Among them, the use of chelating agents is one of the most promising method for removal of metal ions preserving the most meaningful properties of the original soils. One of these methodologies, the Nurchi's method, an extension of the Reilley procedure for EDTA titrations, is based on speciation studies, namely on the knowledge of the related protonation and complex formation constants. Its employment in biomedical and industrial applications is also discussed, namely in the evaluation of the role of different biomolecules such as bacterial metallophores, in metal uptake and homeostasis in living organism (Nurchi et al.).

Determination of organic analytes such as urea or biotoxins in environmental and food samples using novel, advanced and combined analytical techniques was also performed. More in detail, the determination of saxitoxin in seawater samples was made by a novel flow injection microfluidic immunoassay system which allows *in situ* monitoring (Celio et al.). Ultra-trace urea in synthetic and real milk samples was determined by sensitive and selective methods using Fe/N-codoped carbon dots (CDFeN) and a probe with surface-enhanced Raman scattering (SERS), resonance Rayleigh scattering (RRS), and fluorescence (FL) signals (Li C. et al.). Novel methods for the monitoring of microcystins (MCs), one of the most common and harmful cyanotoxins, involve the use of aptasensors (aptamer-based biosensors) and immunosensors (antibody-based biosensors) for rapid, portable, easy-to-use, and on-site determinations (Wang et al.). Early diagnosis of diabetes on entire blood samples was performed *via* near-infrared spectra (NIRS) combined with a support vector machine (SVM) and aquaphotomics (Li Y. et al.).

Quality of milk was assessed by an innovative MicroNIR and chemometric platform for the on-site and contactless monitoring of the samples (Risoluti et al.).

This Research Topic emphasizes the recent methodological improvements made in quantifying and identifying (either explicitly or *via* indirect proxies) those specific chemical species in waters, soil solutions, biological fluids and food products, that provide the most relevant information in human health and environmental safety research. Altogether, these studies point to the wide possibilities underlying the combined use of active/passive sampling strategies, fractionation methods, orthogonal or hyphenated techniques and advanced multivariate analysis for the improvement of the accuracy and specificity of speciation data obtained in complex matrices.

## Author Contributions

All authors wrote the Editorial and reviewed it.

## Conflict of Interest

The authors declare that the research was conducted in the absence of any commercial or financial relationships that could be construed as a potential conflict of interest.
